# Clinical Efficacy of Ultrasound Guidance in Brachial Plexus Nerve Conduction Study: A Comparative Analysis

**DOI:** 10.2174/0115734056377599250717101905

**Published:** 2025-07-24

**Authors:** Zheyuan Zhang, Xiuli Li, Guangju Qi, Huabin Zhang, Xinhong Feng, Zhiyong Bai

**Affiliations:** 1 Department of Ultrasound, Beijing Tsinghua Changgung Hospital, School of Clinical Medicine, Tsinghua University, Beijing 102218, China; 2 Department of Neurology, Beijing Tsinghua Changgung Hospital, School of Clinical Medicine, Tsinghua University, Beijing 102218, China

**Keywords:** Ultrasound guidance, Brachial plexus, Nerve conduction study, Electrodiagnostic testing, Procedural efficiency, Clinical neurophysiology

## Abstract

**Introduction:**

Brachial plexopathy is a diagnostically challenging condition that requires a comprehensive evaluation, including physical examination, imaging, and Electrodiagnostic (EDx).testing. Ultrasound guidance may improve the efficiency and precision of nerve conduction studies by addressing the limitations of blind techniques, such as discomfort and inaccurate localization.

**Methods:**

We prospectively enrolled 30 patients undergoing electrodiagnostic testing. The left upper limb was examined with ultrasound guidance (Group A), while the right upper limb underwent the blind method (Group B). The examined nerves included the median, ulnar, radial, medial and lateral antebrachial cutaneous, axillary, musculocutaneous, suprascapular, and long thoracic nerves. Stimulation duration, number of stimulation attempts, average current, and total examination time were recorded. The differences in data between the two groups were compared and analyzed.

**Results:**

Group A demonstrated significantly lower stimulation duration (156.70±50.13 *vs*. 260.17±53.19 s), fewer stimulation attempts (17.73±3.94 *vs*. 25.80±5.23), and lower average current [32.45 (30.28, 40.13) *vs*. 42.75 (37.78,50.68) mA] compared to Group B (all P < 0.001). No significant difference was observed in total examination time (387.40 ± 33.72 *vs*. 372.00 ± 47.01 s; P = 0.150).

**Discussion:**

Ultrasound guidance improves procedural precision and reduces the need for repeated stimulations and higher electrical intensities. These benefits are achieved without extending the total examination time, making it a feasible and patient-friendly approach for routine use in clinical neurophysiology.

**Conclusion:**

Ultrasound-guided nerve conduction studies of the brachial plexus enhance procedural efficiency and patient comfort compared to the blind method. Further large-scale studies are recommended to validate these findings and assess broader clinical applications.

## 1 INTRODUCTION

Electrodiagnostic (EDx) testing is a crucial diagnostic tool for assessing the function of the neuromuscular system. EDx often includes Nerve Conduction Studies (NCS) and needle electromyography (EMG). EDx plays a significant role in diagnosing peripheral neuropathies, nerve compression syndromes, and other neurological disorders [1, 2]. Among these, brachial plexus examinations are particularly challenging due to the complex anatomy and diverse functional characteristics of the region [3, 4]. Traditional NCS often faces limitations, such as difficulty in accurately locating target nerves, increased patient discomfort, and variability in data quality. These challenges not only compromise the precision of diagnostic results but also negatively impact the patient experience.

Ultrasound guidance plays a crucial role in clinical treatment, particularly in enhancing the accuracy and safety of invasive procedures [5-7]. As a non-invasive, real-time imaging modality, ultrasound enables the precise visualization of target nerves and surrounding anatomical structures [8-10], facilitating accurate needle placement and reducing the risk of blind punctures. This approach has the potential to improve procedural safety, efficiency, and patient comfort. Moreover, by minimizing the need for repeated needle adjustments, ultrasound guidance may reduce pain and enhance the quality of acquired signals. However, systematic investigations comparing the impact of ultrasound guidance with traditional techniques on specific parameters, such as procedural time, discharge frequency, and pain scores, remain limited.

To address this gap, the present study adopts a within-subject design to evaluate the clinical value of ultrasound guidance in NCS of the brachial plexus. One upper limb of each patient was assessed with ultrasound guidance, while the other limb underwent a traditional blind technique. The findings aim to provide evidence-based insights into the utility of ultrasound guidance and inform its broader application in clinical practice.

## 2 MATERIALS AND METHODS

### 2.1 Patients

This study included 30 patients who underwent electrodiagnostic testing at Beijing Tsinghua Changgung Hospital between January and October 2024. The inclusion criteria were as follows: (1) patients presenting with upper limb symptoms suggestive of brachial plexus involvement, such as pain, numbness, or weakness, but finally confirmed to have no brachial plexus-related abnormalities based on their clinical diagnosis, this criterion was set to ensure that each patient could serve as their internal control, (2) patients who consented to participate in the study, and (3) individuals aged 18 to 75 years. Exclusion criteria included: (1) a history of previous brachial plexus surgery or injury, (2) contraindications to NCS procedures or ultrasound-guided interventions, and (3) severe comorbidities that could interfere with the study procedures.

The sample size was estimated based on preliminary data from a pilot study (n = 10), which indicated an effect size of 0.8 for stimulation attempts. A power analysis (G*Power 3.1, α = 0.05, power = 0.8) determined that 26 participants were required; however, 30 were enrolled to account for potential attrition.

All patients underwent an ultrasound-guided examination of the left upper limb (Group A) and a blind method on the right upper limb (Group B). The paired comparison of these two techniques within the same individual minimized variability and allowed for direct assessment of the differences between the methods.

All patients were informed about the study procedures and provided written informed consent prior to participation. This study was approved by the Ethics Committee of Beijing Tsinghua Changgung Hospital.

### 2.2 Instruments and Methods

Ultrasound localization was performed using the DDIT DD70 ultrasound system (China), which is equipped with a 5-10 MHz linear array probe. The probe was used to identify and visualize the brachial plexus and its branches [11, 12], ensuring accurate targeting of the nerves for the subsequent NCS.

**Fig. (1):**
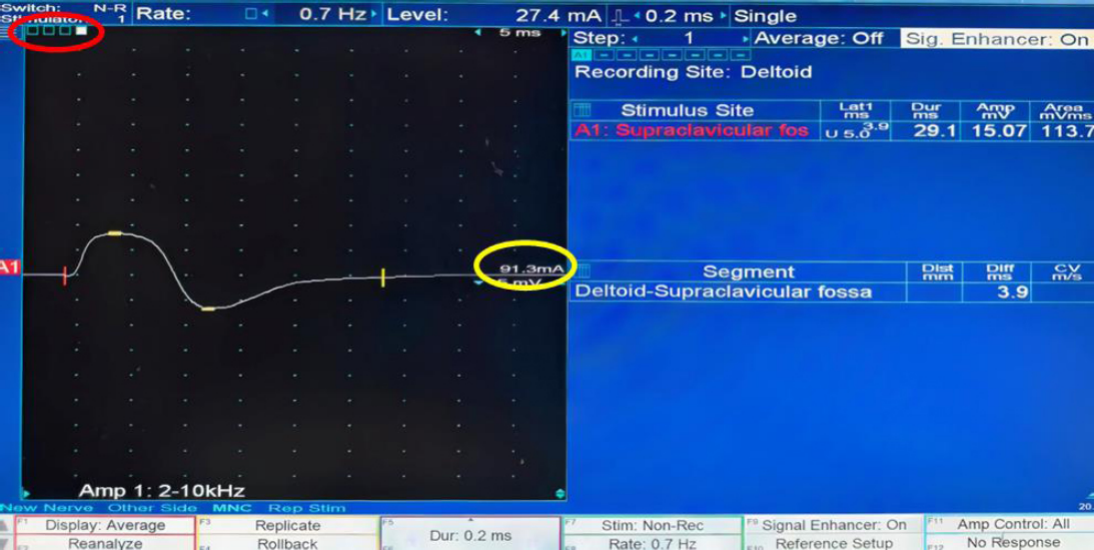
Nerve conduction waveform of the axillary nerve. The red circle highlights the number of stimulation attempts required to obtain the waveform, while the yellow circle indicates the current intensity used to achieve the satisfactory response.

NCS was conducted using the Viking QUEST system (USA). The stimulating electrode was placed in the region of the nerve trajectory, either guided by ultrasound or based on anatomical experience. The recording electrode was positioned on the skin overlying the muscle innervated by the targeted nerve. Electrical stimulation was applied to the nerves, and the position of the stimulating electrode was continuously adjusted to optimize contact with the nerve and ensure effective stimulation. Simultaneously, the current intensity was incrementally modified to achieve appropriate responses without causing excessive patient discomfort. The process continued until two neurologists confirmed that the waveform characteristics met the predefined criteria for proper nerve response (Fig. **1**). If waveforms were suboptimal, further adjustments to electrode placement and stimulation parameters were made.

**Fig. (2):**
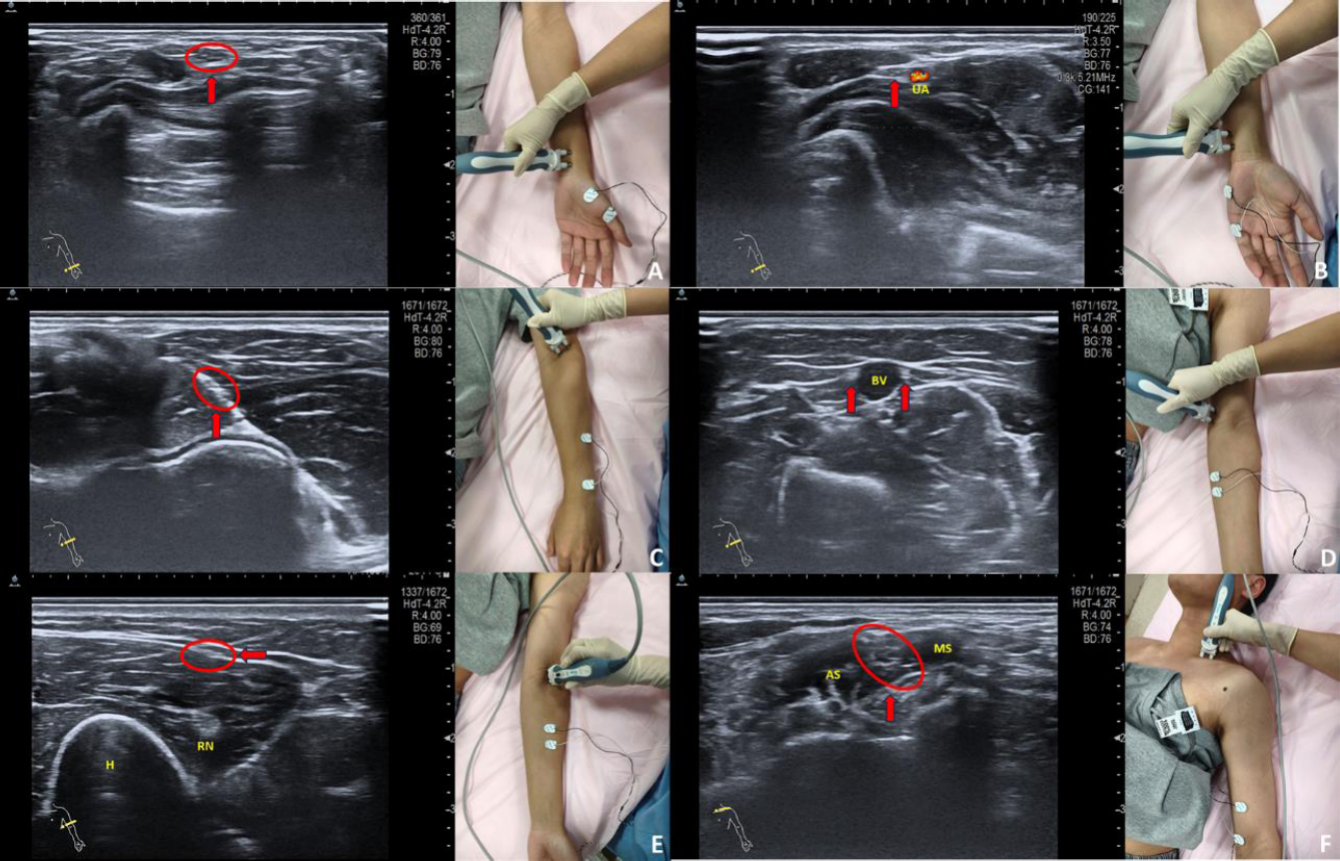
Localization and examination workflow of partial brachial plexus branches. (**A**) Left: Short-axis ultrasound image of median nerve (red arrow) showing its localization; Right: Nerve conduction study of median nerve with the stimulating electrode placed at the identified location. (**B**) Left: Short-axis ultrasound image of ulnar nerve (red arrow) located adjacent to the ulnar artery(UA); Right: Nerve conduction study of ulnar nerve with the stimulating electrode placed at the identified location. (**C**) Left: Short-axis ultrasound image of radial nerve (red arrow) showing its localization; Right: Nerve conduction study of radial nerve with the stimulating electrode placed at the identified location. (**D**) Left: Short-axis ultrasound image of medial antebrachial cutaneous nerve (red arrow) located adjacent to the basilic vein (BV); Right: Nerve conduction study of medial antebrachial cutaneous nerve with the stimulating electrode placed at the identified location.(**E**) Left: Short-axis ultrasound image of lateral antebrachial cutaneous nerve (red arrow) located superficial to the radial nerve (RN) and humerus (H); Right: Nerve conduction study of lateral antebrachial cutaneous nerve with the stimulating electrode placed at the identified location. (**F**) Left: Ultrasound image of interscalene brachial plexus (red arrow), positioned between the anterior scalene (AS) and middle scalene (MS); Right: Nerve conduction study of musculocutaneous nerve with the stimulating electrode placed at Erb’s point.

Fig. (**2**) illustrates the examination workflow for branches of the brachial plexus, including nerve localization using ultrasound and subsequent nerve conduction testing with electrical stimulation. The specific nerves examined included the median nerve (Fig. **2A**), ulnar nerve (Fig. **2B**), radial nerve (Fig. **2C**), medial antebrachial cutaneous nerve (Fig. **2D**), lateral antebrachial cutaneous nerve (Fig. **2E**), musculocuta- neous nerve, axillary nerve, suprascapular nerve, and long thoracic nerve. Notably, the axillary, suprascapular, and long thoracic nerves were localized and stimulated at the same point (Erb's point) as the musculocutaneous nerve (Fig. **2F**), with differences only in the position of the recording electrodes to capture signals from each specific nerve.

Throughout the procedure, several parameters were recorded:

Stimulation Duration: The time for which electrical stimulation was applied during each test.

Number of Stimulation Attempts: The total number of electrical stimulations required to obtain a satisfactory waveform.

Total Examination Time: The time required to complete the entire examination process for one upper limb, including both ultrasound localization and NCS.

Average Current: The average current required to obtain satisfactory waveforms for each nerve during the electrical stimulation.

All procedures for the enrolled patients were performed by the same ultrasound physician and the same neuroelectrophysiology technician to ensure consistency and minimize variability in the results.

### 2.3 Statistical Methods

Data analysis was performed using SPSS 22.0 software (IBM, USA). Continuous variables that followed a normal distribution were described using mean ± Standard Deviation (SD), while variables that did not follow a normal distribution were described using the median and interquartile range [Median (P_25_, P_75_)]. Differences between groups were analyzed using a paired Student’s t-test for normally distributed data and the Mann-Whitney U test for non-normally distributed data. The significance level was set at P < 0.05.

## 3 RESULTS

A total of 30 patients were enrolled in the study, comprising 9 males and 21 females. The mean age of the participants was 53.1±16.0 years, with an age range of 25 to 71 years. Among the 30 patients, 12 (40.0%) reported upper limb pain, 17 patients (56.7%) complained of numbness in the upper limbs, and 15 patients (50.0%) experienced weakness. Following the conclusive clinical diagnosis, the identified causes of these symptoms encompass trauma, muscular disorders, vascular conditions, and cardiovascular diseases. The detailed clinical characteristics of the patients are shown in Table **1**.

The comparison of procedural parameters between Group A (ultrasound-guided) and Group B (blind method) revealed notable differences. The stimulation duration (156.70 ± 50.13 *vs*. 260.17 ± 53.19 s; P < 0.001), number of stimulation attempts (17.73 ± 3.94 *vs*. 25.80 ± 5.23; P < 0.001), and average current [32.45 (30.28, 40.13) *vs*. 42.75 (37.78, 50.68) mA; P < 0.001] were significantly lower in Group A compared to Group B. However, there was no significant difference in the total examination time (387.40 ± 33.72 *vs*. 372.00 ± 47.01 s; P = 0.150) between the two groups, suggesting that the use of ultrasound guidance did not increase the overall procedural duration (Table **2**).

## 4 DISCUSSION

The brachial plexus is a complex network of nerves originating from the cervical and upper thoracic spinal roots (C5–T1), responsible for motor and sensory innervation of the upper limb [13]. Its intricate anatomy and proximity to various anatomical structures make it susceptible to a range of pathological conditions, collectively referred to as brachial plexopathy [14]. Common symptoms of brachial plexus pathology include pain, numbness, and weakness in the upper limb, which can significantly impact patients' daily activities and quality of life. Accurate and efficient diagnosis is crucial for guiding appropriate management and improving clinical outcomes.

Evaluation of brachial plexopathy presents a significant challenge for clinicians. It requires a comprehensive physical examination, various imaging modalities, and EDx to assess the extent of the pathology and determine appropriate treatment strategies [15, 16]. Imaging options include Magnetic Resonance Imaging (MRI), Computed Tomography (CT), and ultrasound. MRI excels at visualizing adjacent soft tissues, while CT is more suited for evaluating potential bony abnormalities. Ultrasound offers distinct advantages, including its non-invasive nature, portability, affordability, real-time imaging capabilities, and the ability to guide related diagnostic or therapeutic interventions [17]. EDx plays a critical role in identifying neuropathology, determining the severity of the condition, and evaluating the distribution of abnormalities. It can also provide insights into the presence of other conditions, such as nerve entrapment syndromes [18]. Despite its high sensitivity and specificity, EDx has notable limitations. It is unable to provide precise anatomical localization of the affected nerves, which can hinder a detailed understanding of the pathology [19]. Additionally, the tests are often uncomfortable and, in some cases, can cause significant pain [20]. Therefore, there is a need for feasible examination methods that not only alleviate the discomfort associated with EDx but also overcome its localization limitations while meeting diagnostic requirements.

This study compared the ultrasound-guided (Group A) and blind (Group B) methods for NCS of the brachial plexus in 30 patients. The results demonstrated that Group A had significantly shorter stimulation duration, fewer stimulation attempts, and reduced average current compared to Group B. These findings indicate that ultrasound guidance improves procedural efficiency and minimizes the intensity of electrical stimulation required to obtain satisfactory waveforms. Interestingly, no significant difference was observed in the total examination time between the two groups. This suggests that while ultrasound guidance enhances certain procedural parameters, it does not add extra time to the overall examination process, making it a feasible option in routine clinical practice. These findings highlight the potential of ultrasound-guided neuroelectrophysiological techniques to improve patient comfort and reduce the burden of nerve stimulation during diagnostic procedures.

The findings of this study can be attributed to the unique advantages of ultrasound guidance in NCS. Ultrasound enables the real-time visualization of nerve anatomy, allowing for the precise localization of target nerves [21]. This accuracy reduces the need for repeated stimulation attempts and minimizes the overall stimulation duration. By identifying the exact position of the nerves, ultrasound guidance also ensures more efficient delivery of electrical currents, resulting in lower average current values required to obtain satisfactory waveforms. In contrast, the blind method relies on anatomical landmarks and palpation [22, 23], which may introduce variability in nerve localization, leading to increased stimulation attempts and longer procedural times. The inability to visualize the nerve may also necessitate higher electrical currents to ensure sufficient stimulation, which can contribute to increased patient discomfort. The lack of significant differences in total examination time between the two methods may be explained by the additional time required for ultrasound setup and scanning, which offsets the time saved by the reduced stimulation attempts. However, this does not detract from the clinical value of ultrasound guidance, as it significantly enhances the accuracy and quality of the examination without prolonging the overall procedure. These mechanistic insights underscore the role of ultrasound as a complementary tool to traditional electrodiagnostic techniques, offering both precision and patient-centered benefits in the evaluation of brachial plexus pathologies.

The results of this study highlight the significant advantages of incorporating ultrasound guidance into NCS of the brachial plexus. By reducing stimulation duration, the number of stimulation attempts, and the average current, ultrasound-guided procedures offer a less invasive and more patient-friendly alternative to the blind method. These benefits are particularly relevant in clinical settings where patient comfort is a priority, such as in pediatric or pain-sensitive populations. Furthermore, the precise anatomical localization provided by ultrasound enhances the accuracy of neurodiagnostic assessments, potentially leading to more reliable identification of brachial plexus pathologies. The ability to visualize adjacent structures in real-time also allows for the detection of anatomical variations or nearby lesions, which may influence both diagnosis and treatment planning [24].

This study has several limitations. First, the sample size was relatively small, and all participants were recruited from a single center, which may limit the generalizability of the findings. Future studies with larger sample sizes and multi-center participation are needed to confirm these results. Second, only patients without brachial plexus abnormalities were included to ensure internal control and comparability, which limits the applicability of the findings to patients with actual nerve pathologies. Third, although procedural time, number of stimulations, and average current were measured, the study did not assess long-term outcomes or potential impacts on diagnostic accuracy. Future research should investigate whether ultrasound guidance improves the diagnostic yield and clinical decision-making in patients with peripheral nerve disorders.

## CONCLUSION

This study demonstrates that ultrasound guidance improves procedural efficiency and patient comfort during NCS of the brachial plexus. By reducing stimulation duration, the number of attempts, and average current, ultrasound guidance offers a valuable enhancement to traditional blind methods, without increasing overall examination time. These findings support the integration of ultrasound into routine electrodiagnostic workflows to improve diagnostic accuracy and patient experience. Future research should focus on larger, multi-center studies to validate these results and explore their application in EMG studies. Ultrasound-guided EMG has the potential to further refine muscle localization, improve signal quality, and expand its utility in assessing neuromuscular disorders. Investigating its role in diverse patient populations and training scenarios will also help solidify its place in clinical practice.

## AUTHORS’ CONTRIBUTIONS

The authors confirm their contributions to the paper as follows: G.Q.: Study conception and design; X.L.: Data collection; Z.Z., X.F., and Z.B.: Drafting the manuscript; H.Z.: Methodology; . All authors reviewed the results and approved the final version of the manuscript.

## Figures and Tables

**Table 1:** Patients’ characteristics.

**30 patients**	-
Age(years)	53.1±16.0
BMI(kg/m^2^)	22.8±2.5
**Gender**	
Male	9(30.0%)
Female	21(70.0%)
**Symptom**	
Pain	12(40.0%)
Numbness	17(56.7%)
Weakness	15(50.0%)
**Cause of symptoms**	
Trauma	8(26.7%)
Muscular disorders	12(40.0%)
Vascular conditions	7(23.3%)
Cardiovascular diseases	3(10.0%)

**Table 2:** Comparison of procedural parameters between the two groups.

-	Group A	Group B	t/Z	P
Stimulation duration(s)	156.70±50.13	260.17±53.19	-6.750	<0.001
Number of stimulation attempts	17.73±3.94	25.80±5.23	-7.753	<0.001
Total examination time(s)	387.40±33.72	372.00±47.01	1.458	0.150
Average current(mA)	32.45(30.28,40.13)	42.75(37.78,50.68)	4.835	<0.001

## Data Availability

The data and supportive information are available within the article.

## LIST OF ABBREVIATIONS

SD: Standard Deviation
EMG: Needle Electromyography
NCS: Nerve Conduction Studies
EDx: Electrodiagnostic Testing

## STANDARDS OF REPORTING

STROBE guidelines were followed.
